# Identification of the Microsporidian *Encephalitozoon cuniculi* as a New Target of the IFNγ-Inducible IRG Resistance System

**DOI:** 10.1371/journal.ppat.1004449

**Published:** 2014-10-30

**Authors:** Marialice da Fonseca Ferreira-da-Silva, Helen Maria Springer-Frauenhoff, Wolfgang Bohne, Jonathan C. Howard

**Affiliations:** 1 Institute for Genetics, University of Cologne, Cologne, Germany; 2 Institute of Medical Microbiology and Hygiene, University of Göttingen, Göttingen, Germany; 3 Instituto Gulbenkian de Ciência, Oeiras, Portugal; 4 Max-Planck Institute for Plant Breeding Research, Cologne, Germany; University of Geneva, Switzerland

## Abstract

The IRG system of IFNγ-inducible GTPases constitutes a powerful resistance mechanism in mice against *Toxoplasma gondii* and two *Chlamydia* strains but not against many other bacteria and protozoa. Why only *T. gondii* and *Chlamydia*? We hypothesized that unusual features of the entry mechanisms and intracellular replicative niches of these two organisms, neither of which resembles a phagosome, might hint at a common principle. We examined another unicellular parasitic organism of mammals, member of an early-diverging group of Fungi, that bypasses the phagocytic mechanism when it enters the host cell: the microsporidian *Encephalitozoon cuniculi*. Consistent with the known susceptibility of IFNγ-deficient mice to *E. cuniculi* infection, we found that IFNγ treatment suppresses meront development and spore formation in mouse fibroblasts *in vitro*, and that this effect is mediated by IRG proteins. The process resembles that previously described in *T. gondii* and *Chlamydia* resistance. Effector (GKS subfamily) IRG proteins accumulate at the parasitophorous vacuole of *E. cuniculi* and the meronts are eliminated. The suppression of *E. cuniculi* growth by IFNγ is completely reversed in cells lacking regulatory (GMS subfamily) IRG proteins, cells that effectively lack all IRG function. In addition IFNγ-induced cells infected with *E. cuniculi* die by necrosis as previously shown for IFNγ-induced cells resisting *T. gondii* infection. Thus the IRG resistance system provides cell-autonomous immunity to specific parasites from three kingdoms of life: protozoa, bacteria and fungi. The phylogenetic divergence of the three organisms whose vacuoles are now known to be involved in IRG-mediated immunity and the non-phagosomal character of the vacuoles themselves strongly suggests that the IRG system is triggered not by the presence of specific parasite components but rather by absence of specific host components on the vacuolar membrane.

## Author Summary

For some time we have studied an intracellular resistance system essential for mice to survive infection with the intracellular protozoan, *Toxoplasma gondii*, that is based on a family of proteins, immunity-related GTPases or IRG proteins. Immediately after the parasite enters a cell, IRG proteins accumulate on the membrane of the vacuole in which the organism resides. Within a few hours the vacuole membrane breaks down and the parasite dies. A puzzle is why this mechanism works on *Toxoplasma*, but only on one other organism among the many tested, namely the bacterial species, *Chlamydia*. What do these widely different parasites have in common that so many other bacteria and protozoa lack? Neither *Toxoplasma* nor *Chlamydia* is taken up by conventional phagocytosis. In this paper we suggest that this is an important clue by showing that a microsporidian, *Encephalitozoon cuniculi*, a highly-divergent fungal parasite, which also invades cells bypassing phagocytosis, is resisted by the IRG system. Therefore, we propose here the “missing self” principle: IRG proteins bind to vacuolar membranes only in the absence of a host derived inhibitor that is present on phagosomal membranes but excluded from the plasma membrane invaginated by IRG target organisms during non-phagosomal entry.

## Introduction

The IFNγ-inducible, immunity-related GTPases (IRG proteins) are a family of proteins essential for innate resistance of mice against certain intracellular pathogens. Until now, the protozoon *Toxoplasma gondii* [1], [2], [3], [4], [5] and its very close relative, *Neospora caninum* [6], [7], as well as two strains of the bacterium *Chlamydia* [8], [9], [10], [11], [12] have been shown to be controlled by the IRG system. However, the IRG resistance system does not engage many other highly diverse organisms, including *Salmonella*, *Listeria*, *Mycobacteria*, *Trypanosoma*, *Rhodococcus* or *Plasmodium*, and the murine-specific strain of *Chlamydia* [reviewed in [13]].

From extended work in the *Toxoplasma* system, we and others have demonstrated that effector IRG proteins such as Irga6, Irgb6, Irgb10, Irgb2-b1 and Irgd relocalise from their cytosolic compartments to the cytosolic face of the parasitophorous vacuolar membrane (PVM) [4], [14], [15] in a GTP-dependent [16], [17] and cooperative manner [18]. In electron microscopy images, the PVM appears ruffled and vesiculated [3], [4], [19]. It is proposed that this action reduces the effective surface-to-volume ratio, putting the PVM under tension against the elastic cytoskeleton of the parasite and leading ultimately to its rupture [13]. Once exposed to the cytosol the parasite dies for unexplained reasons [3], [4], [20].

IRG proteins can be divided into the effector GKS subfamily, including the IRGA and IRGB proteins and Irgd (all carrying a canonical GxxxxGKS motif in the P-loop of the GTP-binding site) and the regulatory GMS subfamily, namely Irgm1, Irgm2 and Irgm3 (with a non-canonical GxxxxGMS motif) [21], [22]. GMS proteins populate the vacuoles of *T. gondii* or inclusions of *Chlamydia* either not at all (Irgm1) or only to a limited extent (Irgm2, Irgm3) [1], [4], [18], [23]; their function is to inhibit inappropriate GTP-dependent activation of the GKS proteins on host vesicular compartments in IFNγ-induced cells before the parasite enters [16].

It is not known why the action of IRG proteins is restricted to so few and so dissimilar parasitic organisms. We hypothesized that unusual features of the intracellular replicative niches of *T. gondii* and *Chlamydia* strains, neither of which resembles a phagosome, might hint at a common principle. To test this hypothesis we decided to examine cell-autonomous resistance *in vitro* to the microsporidian, *Encephalitozoon cuniculi*. The Microsporidia, recently re-classified as the earliest divergent group of the Fungi [24], [25], are abundant, obligate intracellular eukaryotic parasites of many diverse animal groups including mammals. *E. cuniculi* is a convenient representative since it is easily cultivated *in vitro* and its genome is fully sequenced, which is at 2.9 Mb one of the smallest known eukaryotic genomes [26]. *E. cuniculi* and its relatives *E. hellem* and *E. intestinalis* are common opportunistic pathogens for immunocompromised humans [27].

Microsporidia, including *E. cuniculi*, have a peculiar entry mechanism into host cells utterly unlike conventional phagocytosis. The thick-walled unicellular spore of *E. cuniculi* contains the organism itself (the sporoplasm) and a coiled proteinaceous tube, the polar tube, which can be suddenly extruded as a result of an osmotic stimulus and pushes a deep and narrow invagination in any adjacent host cell plasma membrane. The sporoplasm is then expelled through the polar tube and can be found deep in the host cytoplasm in an intracellular parasitophorous vacuole bounded by a membrane mainly derived from the host plasma membrane [reviewed in [28]]. The intracellular sporoplasm, now termed meront, divides repeatedly in its vacuole and eventually differentiates into large numbers of spores, finally lysing the host cell to release the mature environmentally-resistant spores [29]. By virtue of its remarkable non-phagocytic entry mechanism, this natural parasite of rodents and rabbits was an interesting potential target for the IRG resistance system, and it has been reported that IFNγ induces strong cell-autonomous immunity against this organism [30], [31], [32].

In the present study we show that the IRG system is indeed required for cell-autonomous resistance to *E. cuniculi*. We confirm the development of resistance following IFNγ treatment of fibroblasts and show that several IRG proteins localise to the PVM of intracellular *E. cuniculi*. Moreover, *E. cuniculi* infection triggered IFNγ-dependent reactive cell death, as seen earlier in *T. gondii* resistance [20]. To demonstrate the importance of IRG proteins in IFNγ-dependent growth restriction of *E. cuniculi*, we show that IFNγ-mediated resistance was completely lost in mouse cells that are deficient in the regulatory GMS proteins, Irgm1 and Irgm3, a double deficiency that inactivates the whole IRG system.

The phylogenetic range of the three classes of target organism of the IRG resistance system - bacteria, a fungus and protozoa - and their vastly dissimilar biology, strongly suggests that the specificity with which IRG proteins localise to parasitophorous vacuoles relates to a common characteristic of the host-derived membrane of such vacuoles rather than to a common ligand derived from the parasites themselves.

## Results

### IFNγ restricts *E. cuniculi* growth in primary mouse fibroblasts

It was first reported in 1995 that IFNγ induction restricts microsporidial growth in mammalian cells *in vitro* using *E. cuniculi* infection of murine peritoneal macrophages [30]. Subsequent studies confirmed the suppressive effect of IFNγ on *E. cuniculi* growth as well as on *E. intestinalis* using murine peritoneal macrophages [31], [32], the murine enterocyte cell line CMT-93 and human enterocyte cell line Caco-2 [33] as well as primary human monocyte-derived macrophages [34]. Furthermore, IFNγ-deficient mice are susceptible to *E. cuniculi* and *E. intestinalis* infection [32], [35], [36], [37]. It is characteristic of the IRG resistance system that it is efficient in non-myeloid cells such as fibroblasts [1], [4], [38]. We therefore tested IFNγ-dependence of cell-autonomous resistance against *E. cuniculi* in primary mouse embryonic fibroblasts. IFNγ-induced and uninduced C57BL/6 mouse embryonic fibroblast (MEF) monolayers were infected with *E. cuniculi* spores and the replication of the parasites followed by immunofluorescence microscopy and Western Blot analysis. *E. cuniculi* was detected with an antibody directed against a cytoplasmic protein (mAB 6G2) of the earliest infectious stages (meronts), or an antiserum against the spore wall protein 1 (pAS anti-SWP1), which is synthesized later in infection [39].

**Figure 1:**
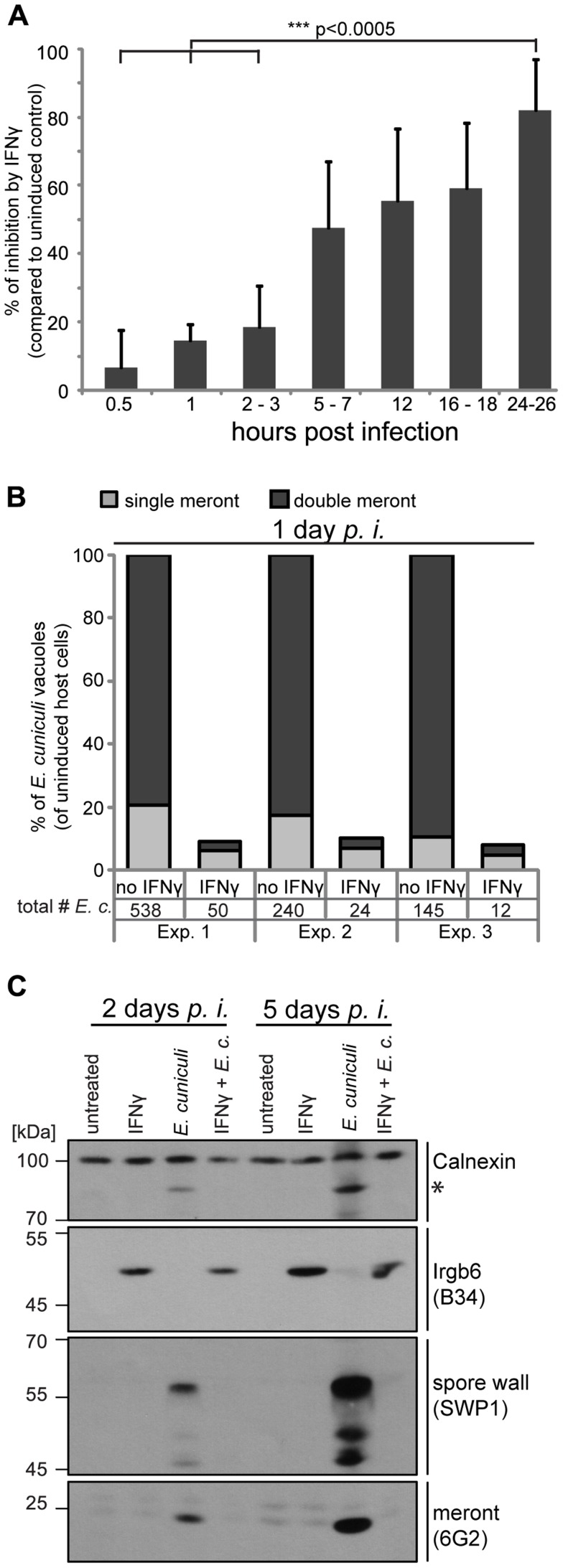
IFNγ restricts *E. cuniculi* growth in mouse embryonic fibroblasts. (**A**) Mouse embryonic fibroblasts (MEFs) from C57BL/6 mice were induced with IFNγ for 24 h or left uninduced before infection with *E. cuniculi* spores. Cells were fixed at the indicated time points and the number of meronts (stained with anti-meront mAb 6G2) per 500 host nuclei (stained with DAPI) was counted. The inhibition in the IFNγ-treated sample compared to the uninduced control sample is presented as mean +/− standard deviation (SD) of 3–7 replicates per time point from at least 2 individual experiments. Significant differences (of 0.5 h, 1 h and 2–3 h compared to 24–26 h) were calculated with a two tailed T-test. (**B**) MEFs were induced with IFNγ or left uninduced, infected with *E. cuniculi* spores for 24 h and stained as in A. Single meronts and meronts that divided once (double meront) were counted per 500 host nuclei and shown as percent of total vacuoles of uninduced controls. Numbers indicate the counted number of single or double meronts per 500 host cells. Data from three independent experiments (Exp. 1–3) is presented. (**C**) MEFs were stimulated with IFNγ and/or infected with *E. cuniculi* spores for 2 or 5 days or left untreated. Cell lysates were separated by SDS-PAGE and Western Blots were cut into three regions to probe for anti-meront mAB 6G2 as well as anti-spore wall protein 1 pAS SWP1. Calnexin staining served as loading control and Irgb6 staining (mAB B34) to proof IFNγ-induction. The asterisk marks an unknown *E. cuniculi*-derived protein that is detected by the Calnexin antibody. These Western Blots emerged from one single SDS-PAGE, the 45–70 kDa region was first probed with mouse mAB B34, stripped, and then probed for anti-SWP1 rabbit pAS. Experiments for both time points were performed at least three times.

A time series of 0.5 to 24 hours post infection showed continuous IFNγ-dependent loss of *E. cuniculi* meronts (determined by counting of 6G2-positive meronts per host nuclei). Meront numbers at the earliest time point measured (0.5 hours) were equivalent in IFNγ-treated and untreated cells showing that *E. cuniculi* invasion into the host cells was not significantly affected by prior IFNγ induction (Figure 1A). Next, we quantified not only single meronts, but also meronts that had replicated by binary fission (double meronts) and compared uninduced to IFNγ-induced MEF cells 24 hours post infection. Of the few surviving meronts at 24 h, very few had successfully divided in IFNγ-induced cells (Figure 1B).

In addition, Western Blot analysis of whole cell lysates from infected MEF cells showed that meront development as well as the formation of new spores was blocked by IFNγ (Figure 1C). In uninduced MEF cells, *E. cuniculi*-dependent protein bands were detected with the meront-specific antibody, indicative of replication, and with the spore-specific antiserum, indicative of maturation at 2 days post infection. The intensity of these parasite-specific bands further increased at 5 days post infection. In contrast, these bands could not be detected in *E. cuniculi*-infected IFNγ-induced cells either 2 or 5 days after infection. Taken together, IFNγ kills meronts, inhibits meront replication, and blocks spore formation of *E. cuniculi* in primary mouse embryonic fibroblasts.

### IRG proteins accumulate on the PVM of *E. cuniculi*

**Figure 2:**
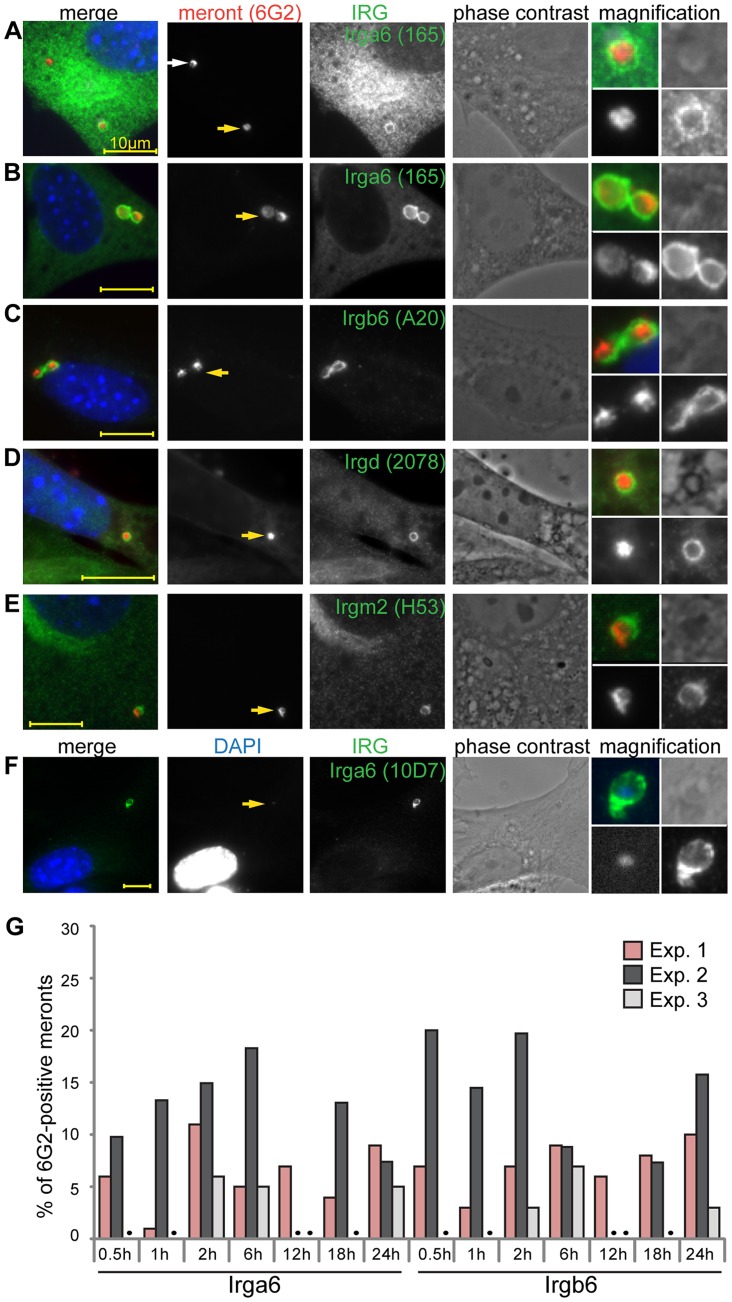
IRG proteins accumulate at the *E. cuniculi* parasitophorous vacuolar membrane. (**A–F**) MEFs were induced with IFNγ for 24 h and then infected with *E. cuniculi* spores for 24 h. Fixed cells were stained for anti-meront mAB 6G2 as well as for endogenous Irga6 (165/3 pAS and 10D7 mAB), Irgb6 (A20 pAB), Irgd (2078 pAS) and Irgm2 (H53 pAS). Nuclei were labeled with DAPI. Representative microscopic images of IRG-positive *E. cuniculi* PVMs are presented. Yellow arrows point at the IRG-loaded PVM which is magnified at the end of each panel (zoom in the following order: upper left: merged image, upper right: phase contrast, lower left: anti-meront, lower right anti-IRG); white arrow: unloaded meront, every scale bar is 10 µm. (**G**) Quantification of Irga6 and Irgb6 loading onto the *E. cuniculi* PVM at different time points post infection. 100 vacuoles were evaluated per sample, a black dot indicates that sample was not counted, three independent experiments are shown.

When *T. gondii* infects IFNγ-treated mouse fibroblasts, the induced IRG proteins, especially the effector GKS proteins, accumulate on the PVM and lead to disruption of the vacuole [20]. To examine whether similar IRG-related processes might also occur on the microsporidian vacuole, we co-stained IFNγ-treated, *E. cuniculi*-infected, MEF cells with immunological reagents against individual GKS effector proteins (Irga6, Irgb6 and Irgd) as well against the GMS regulator proteins (Irgm1 and Irgm2) 24 h post infection (Figure 2). Some meronts were indeed coated with IRG proteins, but most were IRG-negative. Both Irga6-coated and uncoated vacuoles were found together in multiply infected host cells (Figure 2A). Irga6 and Irgb6 were found on vacuoles at higher frequencies, while Irgd and Irgm2 were found at lower but consistent frequencies (below 5%) (Figure 2B–E). Irgm1 was never found at the *E. cuniculi* PVM (more than 1000 meronts in three independent experiments were analysed). In IFNγ-induced cells, cytoplasmic Irga6 is predominantly in the GDP-bound form, but accumulates on the *T. gondii* PVM in the GTP-bound activated form, which can be specifically detected with the mouse antibody10D7 [17]. Because we could not conduct a co-staining with the mouse anti-meront antibody, 6G2, in combination with 10D7, we identified the meront via its enhanced DAPI signal to show that indeed Irga6 was accumulating on *E. cuniculi* vacuoles in the GTP-bound state (Figure 2F) as in *T. gondii* immunity. The number of vacuoles positive for Irga6 or Irgb6 was examined in more detail at different time points after infection (Figure 2G). The frequency of Irga6- and Irgb6-positive vacuoles varied between experiments (1–20%), but did not significantly increase or decrease between 0.5–24 hours post infection (Figure 2G) as the number of meronts progressively dropped, suggesting relatively fast clearance of the IRG-positive vacuoles.

### IRG proteins load onto PVM of *E. cuniculi* in a cooperative manner

**Figure 3:**
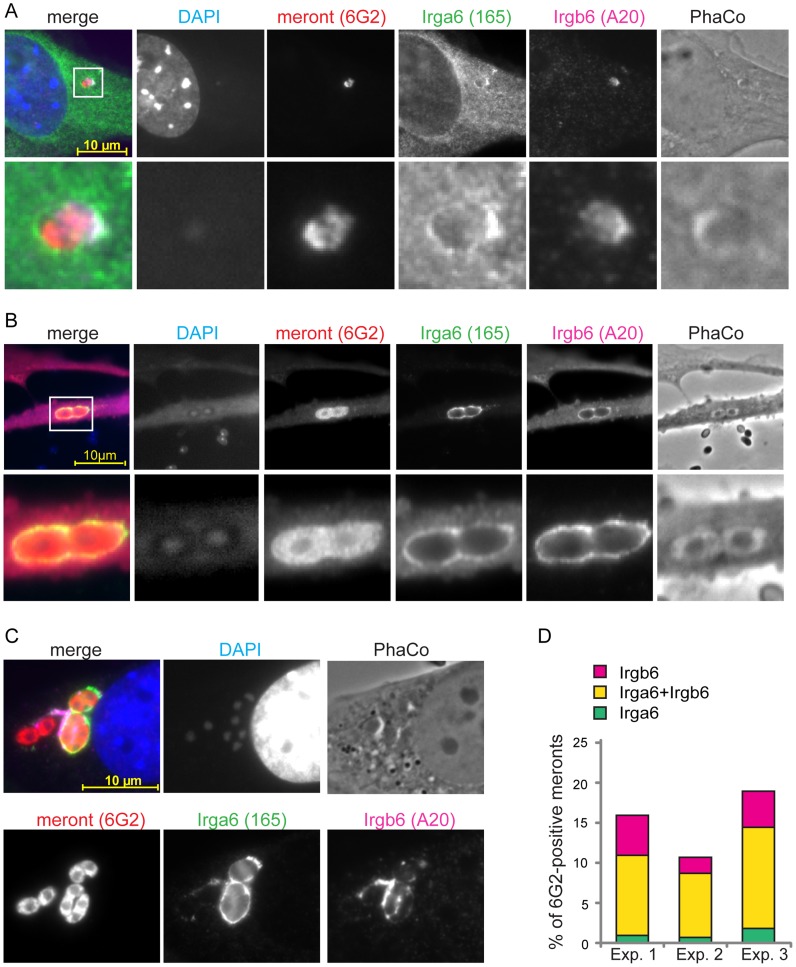
IRG proteins load onto PVM in a cooperative manner. MEFs were induced with IFNγ for 24 h and then infected with *E. cuniculi* spores for 12 h (**A**) or 24 h (**B, C**). Fixed cells were stained for meronts (mouse mAB 6G2, red) as well as for endogenous Irga6 (rabbit pAS165/3, green) and Irgb6 (goat pAB A20, far-red pseudocolored in magenta). Nuclei were labeled with DAPI. Representative images from 4 independent experiments are shown; white box indicates enlarged area shown below; scale bar: 10 µm. (**D**) Quantification of cooperative loading after 24 h; Irgb6-single, Irga6-single or Irgb6/Irga6-double (both) positive meronts are shown as % of total 6G2-positive meronts; 100 vacuoles were counted in each independent experiment (Exp. 1–3).

A detailed view of IRG loading onto the *T. gondii* PVM has been established. IRG proteins accumulate on the PVM in a hierarchical order with Irgb6, Irgb10 and Irga6 as pioneers and demonstrate cooperative behaviour by stabilizing each other at the PVM [18]. In order to investigate cooperative loading on the *E. cuniculi* PVM, we conducted triple immunofluorescent stainings to identify the meront and two GKS proteins, Irga6 and Irgb6. Individual vacuoles positive for both IRG proteins were observed at early and late time points, such as 12 h post infection (Figure 3A) and 24 h post infection (Figure 3B, C). In most cases, Irga6 and Irgb6 co-localised to single vacuoles (Figure 3B), although often without accurate spatial coincidence on the PVM (Figure 3C). Notably, the number of *E. cuniculi* vacuoles accumulating both IRG proteins was higher than single-coated ones (Figure 3D). In view of the low frequencies of accumulation of individual IRG proteins, it is clear that the frequency of double-loaded vacuoles is highly non-random, suggesting that loading is cooperative between different IRG proteins, as seen on the *T. gondii* PVM.

### IFNγ-mediated inhibitory effects on *E. cuniculi* are lost in GMS-deficient cells

**Figure 4:**
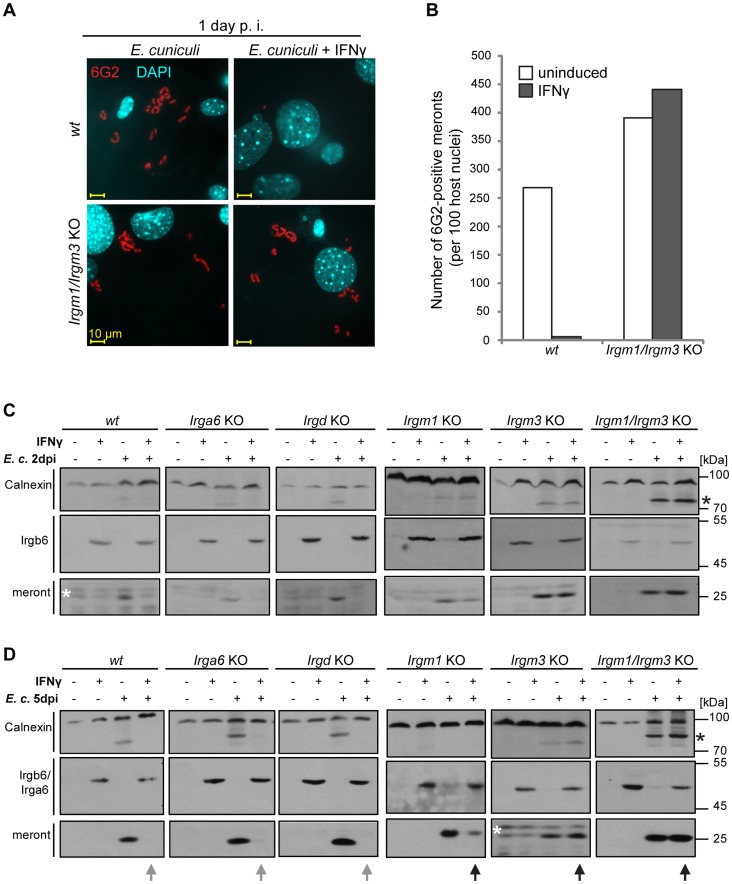
IFNγ suppressive effect on *E. cuniculi* growth is impaired in GMS-IRG knock-out cells. (**A**) *Wildtype (wt)* or *Irgm1/Irgm3* knock-out (KO) MEFs were induced with 200 U/ml IFNγ for 24 h and then infected with *E. cuniculi* spores for 24 h or left untreated. Cells were fixed and stained for meronts using 6G2 mAB (red) and host nuclei with DAPI (pseudocolored in cyan). Representative fluorescence microscopic images are shown. (B) Quantification of A, representative of two independent experiments. (C/D) Transformed *wildtype* or transformed IRG knock-out MEFs were induced with IFNγ for 24 h and then infected with *E. cuniculi* spores or left untreated. Cells were harvested after 2 days (in D) and 5 days (in E) post-infection. Cell lysates were separated by SDS-PAGE and Western blots were cut into three regions and simultaneously probed for anti-meront mAB 6G2, anti-Calnexin pAB, which served as loading control and anti-Irgb6 (mAB B34) or anti-Irga6 (mAB 10E7 for *Irgm1* KO and *Irgm3* KO MEFs both at 5 d post infection; 165/3 pAS for *Irgm1/Irgm3*KO MEFs at 5 d post infection) as IFNγ-induction control. The black arrows highlight a 6G2-positive protein band indicating *E. cuniculi* growth despite presence of IFNγ, which is inhibited in *wt* cells (grew arrows). The asterisk marks an unknown *E. cuniculi*-derived protein that is detected by the Calnexin antibody. The white asterisk marks unspecific bands. The four samples of one cell line per time point were analyzed together by one single SDS-PAGE and Western Blot, except for the *Irgm1*KO MEFs at 2 d post infection. The data represents at least three independent experiments.

To assess the importance of IRG proteins in the IFNγ-dependent restriction of *E. cuniculi*, we investigated the development of the parasite in cells derived from *IRG* knock-out mice. First, we examined *E. cuniculi* infection in IFNγ-induced primary *wildtype* and *Irgm1/Irgm3* ^−/−^ MEF cells, which lack the two regulator GMS proteins, Irgm1 and Irgm3, and also express reduced levels of GKS proteins [40]. The number of meronts observed in *Irgm1/Irgm3* ^−/−^ MEF cells 24 h after infection was the same whether the cells were induced with IFNγ or not (Figure 4A, B). In contrast, and as observed in Figure 1, the number of meronts in IFNγ-induced *wildtype* cells was drastically reduced at 24 h after infection compared with uninduced controls (Figure 4A, B).

We next assayed IFNγ-inducible resistance to *E. cuniculi* in transformed fibroblasts from mice deficient in single IRG genes as well as in the *Irgm1/Irgm3* ^−/−^ double knock-out cells. Parasite growth was assessed with the anti-meront antibody by Western blot, while the expression of Irgb6 (or Irga6) confirmed successful IFNγ induction (Figure 4C–D). In *wildtype* cells, IFNγ-induction resulted in complete loss of the meront marker at 2 and 5 days after infection, while in the *Irgm1/Irgm3* ^−/−^ double knock-out cells IFNγ-induction caused no inhibition of meront growth. Single mutants for either *Irga6* or *Irgd*, two members of the GKS effector subfamily, showed no loss of resistance relative to *wildtype* cells. However, cells lacking one GMS protein, *Irgm1* or *Irgm3*, both showed clear susceptibility phenotypes. Susceptibility of the *Irgm1*-deficient cells was incomplete, while *Irgm3*-deficient cells were apparently as susceptible as *Irgm1/Irgm3* double-deficient cells. Interestingly, Irgm3 deficiency also has a stronger susceptibility phenotype than Irgm1 deficiency for *T. gondii* in IFNγ-induced MEFs (unpublished observations). The stronger phenotype from the GMS knock-outs is expected, because these deficiencies deregulate all the GKS effectors [41]. The undetectable effects of the two GKS effector knock-outs is consistent with the much weaker *in vivo* phenotypes of single Irga6 and Irgd deficiencies in *T. gondii* infection [2], [42]. A deficiency of several GKS effectors would be expected to show a stronger phenotype.

### *E. cuniculi* infection triggers IFNγ-dependent host cell death

**Figure 5:**
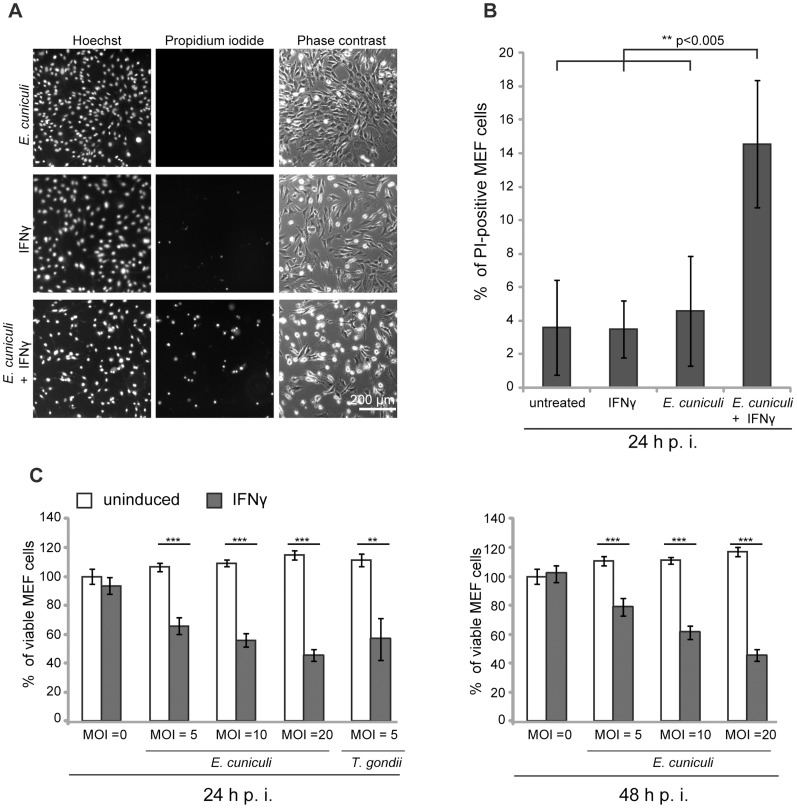
*E. cuniculi* infection triggers IFNγ-dependent host cell death. (**A**) *Wt* MEFs were induced with IFNγ for 24 h and then infected with *E. cuniculi* spores for 24 h or left untreated. Without fixation, the cells were stained with Hoechst and Propidium iodide dye, photographed under live-cell conditions and automatically enumerated. (**B**) Quantification of A, graph represents mean values +/− SD from five independent experiments. (**C**) *Wt* MEFs were seeded in 96-wells and induced with IFNγ for 24 h (black bars) or left untreated (white bars). Cells were infected with *E. cuniculi* spores at different MOIs (MOI = 5–20) or with *T. gondii* Me49 tachyzoites (MOI = 5) as positive control. Cell viability was measured with a formazan-based colorimetric assay 24 h or 48 h post infection and expressed as percentages of uninduced uninfected control cells. Graph represents mean value +/− SD of triplicates of one representative experiment. Three independent experiments were performed; Significance was calculated with two-tailed T-Test: ** p>0.005, *** p>0.0005.

We and others have documented the direct disruption of the IRG protein-coated *T. gondii* PVM, followed by necrotic host cell death in mouse cells induced by IFNγ [4], [15], [20], [43]. It was of interest to find out whether this consequence of IRG protein action could also be observed in IFNγ-induced mouse cells infected with *E. cuniculi*. In the first experiments we stained infected primary MEF *wildtype* cells under live-cell conditions with the membrane-impermeable dye propidium iodide in order to stain nuclei of necrotic cells, and with the membrane–permeable dye Hoechst, which stains all nuclei. We found a significant excess of propidium iodide-positive nuclei in *E. cuniculi* infected and IFNγ-treated cells compared to untreated or single-treated control samples (Figure 5A, B). Next, we used a standard formazan-based colorimetric assay to measure viability of MEF *wildtype* cells with increasing multiplicity of infection with *E. cuniculi*. At one day post infection, viability of infected cells was significantly reduced in dependence of IFNγ-induction and this was even more pronounced at 2 days post infection (Figure 5C). Thus, in the presence of IFNγ, *E. cuniculi* infection seems to induce the same response as *T. gondii*, namely reactive death of the host cell itself.

### IDO is not responsible for IFNγ-mediated *E. cuniculi* restriction

**Figure 6:**
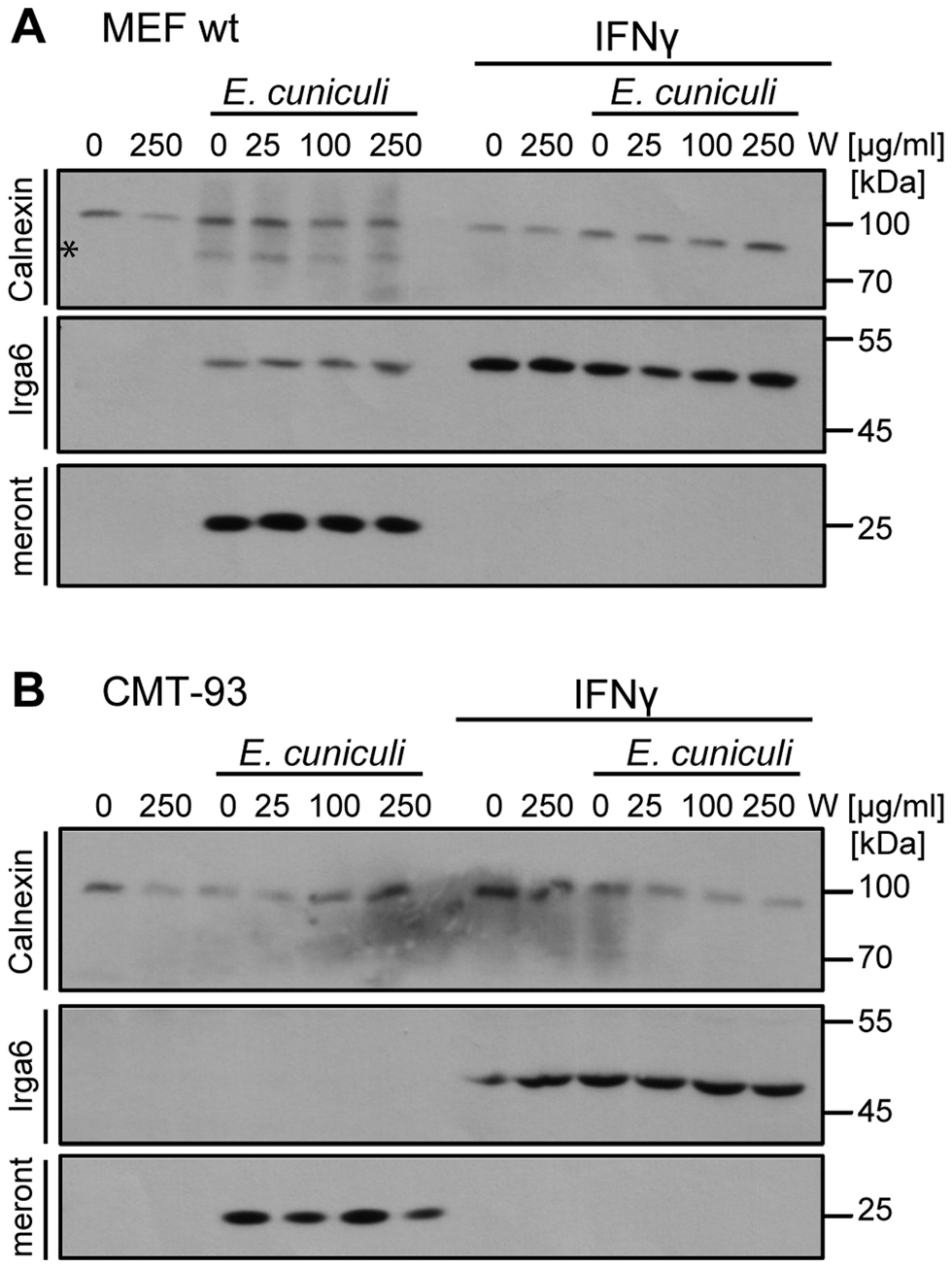
Tryptophan supplementation cannot reverse the IFNγ-mediated *E. cuniculi* restriction. C57/BL/6 MEFs (**A**) or mouse enterocytic CMT-93 cells (**B**) were treated with IFNγ for 24 h or left uninduced. Indicated doses of L-Tryptophan (W) were added to the medium 30 minutes prior infection with *E. cuniculi* spores. Cell lysates were prepared 5 days post infection and separated by one SDS-PAGE. Western blots were cut into three regions probed simultaneously with for anti-meront mAB 6G2, anti-Calnexin pAB as loading control and anti-Irga6 (10E7 mAB) as IFNγ-induction control. The data are representative of three independent experiments.

Restricting nutrient acquisition is a common defence mechanism against intracellular parasites. Deprivation of tryptophan by the IFN-inducible indoleamine 2,3-dioxygenase (IDO) is often claimed to be the main inhibitor of *T. gondii* replication in IFNγ-induced human fibroblasts [reviewed in [44], [45]], following reports that replication can be rescued by supplementation of the medium with tryptophan [46], [47]. IDO-mediated growth restriction of *E. intestinalis* has been proposed following observations in a mouse enterocytic cell line CMT-93 [33]. However, another study in activated mouse peritoneal macrophages showed that L-tryptophan supplementation failed to rescue the infection [48]. In view of these apparently inconsistent results, we analysed *E. cuniculi* growth in IFNγ-induced mouse cells following tryptophan supplementation (Figure 6). In *wildtype* MEF cells, as well as in CMT-93 cells, IFNγ-mediated growth restriction on *E. cuniculi* could not be reversed by supplementation with excess tryptophan, arguing strongly against mediation of the inhibition via IDO. Taken together with the complete loss of resistance caused by IRG protein deficiencies, we conclude that the IFNγ-mediated restriction of *E. cuniculi* in non-myeloid cells is mediated exclusively by the IRG system in mice.

## Discussion

The IFN-inducible IRG proteins of the mouse are essential for resistance against some strains of the intracellular bacterium, *Chlamydia*, and against the intracellular protozoon, *Toxoplasma gondii*, but seem to play no role in resistance against a multitude of other intracellular bacterial and protozoal infections. The combination of selectivity and lack of phylogenetic consistency in IRG protein action calls for a mechanistic explanation that unifies the two widely disparate target species while excluding organisms that do not engage the IRG system. The purpose of this study was to test the hypothesis that the key lies in how different organisms enter the host cell. Most of the organisms that are ignored by the IRG system, such as *Salmonella*, *Listeria*, *Leishmania*, *Mycobacteria*, and *Rhodococcus*, engage the phagocytic mechanism and are taken up into and reside, whether temporarily or permanently, in more or less modified phagosomes. In contrast *T. gondii* typically enters actively using force generated by its own cortical cytoskeleton without engaging the phagocytic mechanism [reviewed in [49], [50]]. However, it has recently been described that *T. gondii* may enter macrophages, but not fibroblasts, by phagocytic uptake, but this is followed by active exit from the phagosome into a conventional parasitophorous vacuole and the loading of Irgb6 appears to be unaffected [51]. *Chlamydia* is taken up by an unknown process with some features of clathrin-mediated endocytosis but none of phagocytosis [52]. With this disparity in mind, the working hypothesis behind this paper was that organisms that enter cells without engaging the phagocytic mechanism may become preferential targets for IRG protein-mediated resistance, regardless of their taxonomic status. To generalise this idea, we tested another intracellular organism with an anomalous, non-phagocytic mode of cellular invasion and a wide taxonomic divergence from the other two known IRG protein targets: the microsporidian, *Encephalitozoon cuniculi*.

The unambiguous conclusion from our experiments is that infection by *E. cuniculi* is resisted in IFNγ-induced mouse fibroblasts by the action of the IRG proteins, and several aspects of the process closely resemble features that have been studied in detail in *T. gondii* infection. Resistance is associated with accumulation of IRG proteins onto at least a proportion of the intracellular organisms within the first 30 minutes after infection, a time at which IRG protein accumulation onto the *T. gondii* vacuolar membrane is already well-advanced [18]. At the light microscopical level it is not possible to say precisely where the IRG proteins are localised, but images from later time points after infection, when the vacuole is enlarged and the PVM is separated from the organism by an intravacuolar space, suggest that the IRG proteins are loaded onto the parasitophorous vacuole membrane.

**Figure 7:**
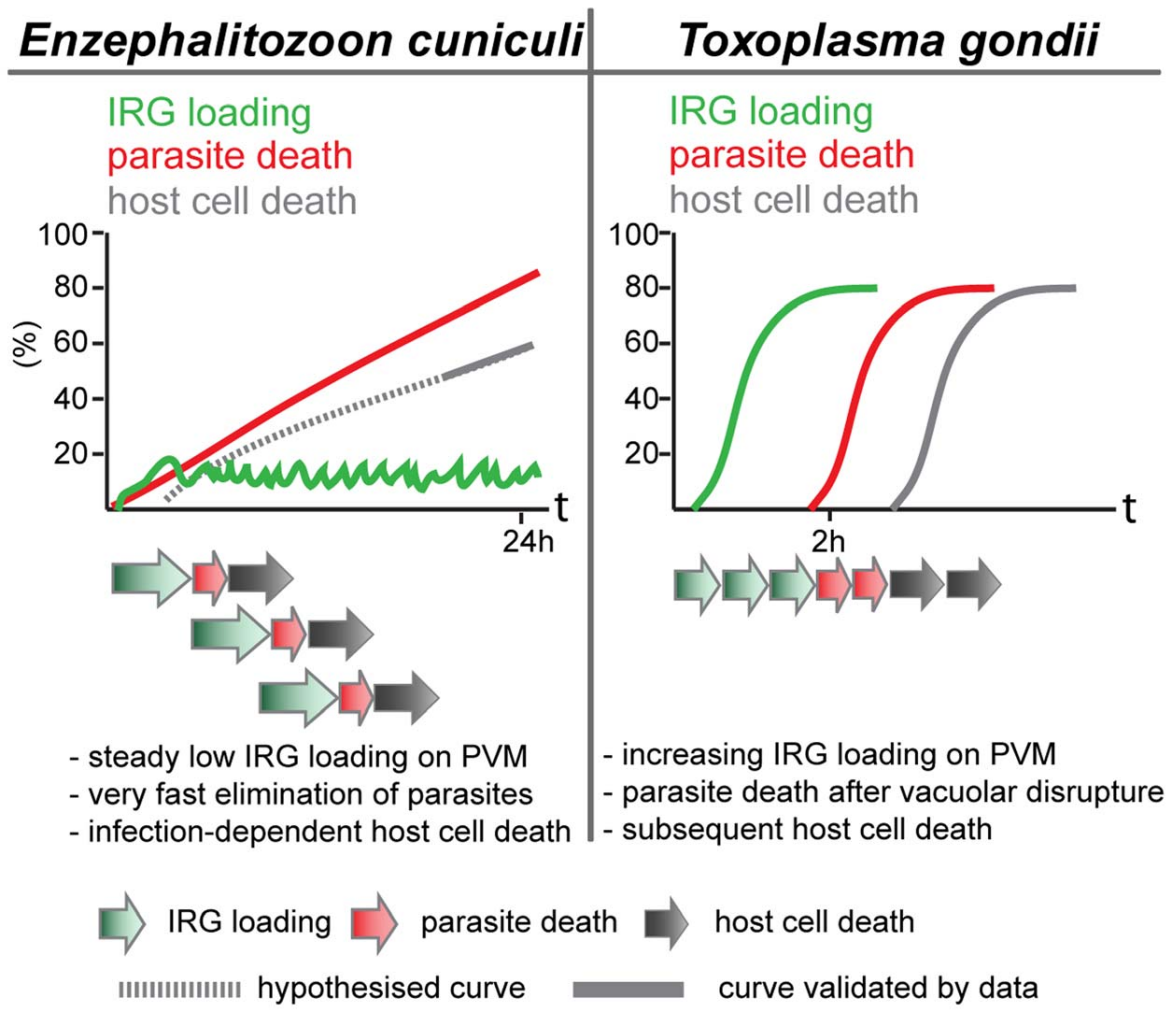
Scheme of different dynamics of IRG action. Upon *E. cuniculi* infection, a low but steady number of IRG-positive vacuoles can be detected over the first 24 h post infection accompanied by a continuous loss of viable meronts. Moreover, host cell death is triggered by the combination of *E. cuniculi* infection and IFNγ induction. Initiation of IRG loading might be a stochastic and asynchronous event that is followed by a rapid elimination of the pathogen. In contrast, IRG loading on PVs of avirulent *T. gondii* strains starts immediately after parasite invasion. IRG-positive vacuoles seem to accumulate reaching a maximum at about 2 h post infection. When fully loaded, the vacuoles disrupt followed by *T. gondii* death and host cell death in an invariant order.

The cooperative pattern of loading of the different IRG proteins is also familiar from *T. gondii*. The frequency of vacuoles loaded at any time is low, but the majority of vacuoles carry more than one IRG protein (Figure 3). This result could of course also arise if only a few vacuoles are receptive to IRG proteins at any time. However, data from *T. gondii* showed that the loading of Irgb6 was stabilised and enhanced by the loading of Irga6, and thus clearly cooperative [18]. There is also a tendency in the *E. cuniculi* infection, perhaps not so well marked as in *T. gondii* infection, for Irgb6 to load more vacuoles than Irga6, and Irgd to load fewer. Also, as in *T. gondii* [1], [4] and in *C. trachomatis* infection [8], [10], [23], the IRG regulatory protein, Irgm1, does not load onto any *E. cuniculi* vacuoles, while Irgm2 can be found on some. In another respect, however, the loading of *E. cuniculi* vacuoles with IRG proteins appears to be different from the loading of *T. gondii* vacuoles. With avirulent *T. gondii*, the number of vacuoles loaded with IRG proteins rises to as much as 90% of all vacuoles within 2 h after infection. With *E. cuniculi*, the number of vacuoles loaded reaches a plateau between 5 and 15% within 30 minutes of infection, and persists at that level for many hours while the number of live meronts progressively falls (Figure 2). These different loading behaviours can be reconciled with qualitatively similar processes operating on the vacuoles of both organisms, if the initiation of IRG protein loading onto individual *E. cuniculi* vacuoles takes on average longer than onto *T. gondii* vacuoles, and if *E. cuniculi* vacuoles subsequently disintegrate and are cleared with faster kinetics than *T. gondii* vacuoles. With increasing time after infection more and more parasites are cleared from the cells, accounting for the long, slow loss of detectable meronts (Figure 1) reaching about 90% only 24 h after infection (Figure 7).

Light microscopy does not allow us to see exactly what happens to *E. cuniculi* vacuoles after loading with IRG proteins. Clear-cut disruption typical of the IRG-loaded *T. gondii* vacuole [20] is not easy to register. Nevertheless the vacuoles and their included parasites disappear (see Figure S1). In *T. gondii*, the disruption of the vacuole is followed within about 20 minutes by the death of the parasite and after an hour or two by the necrotic death of the infected cell. We also observed an excess of dead, presumably necrotic, cells in IFNγ-induced fibroblasts infected with *E. cuniculi*.

Lastly, as with *T. gondii* resistance, the IRG system appears to be the only mechanism in IFNγ-induced mouse fibroblasts that is capable of restriction of *E. cuniculi*. In fibroblasts from mice double deficient for the regulator IRG proteins, Irgm1 and Irgm3, in which the whole IRG system is largely disabled, all IFNγ-inducible resistance against the growth and development of the parasite was lost, and the IFNγ-inducible catabolic enzyme for tryptophan played no role in resistance against *E. cuniculi*.

**Figure 8:**
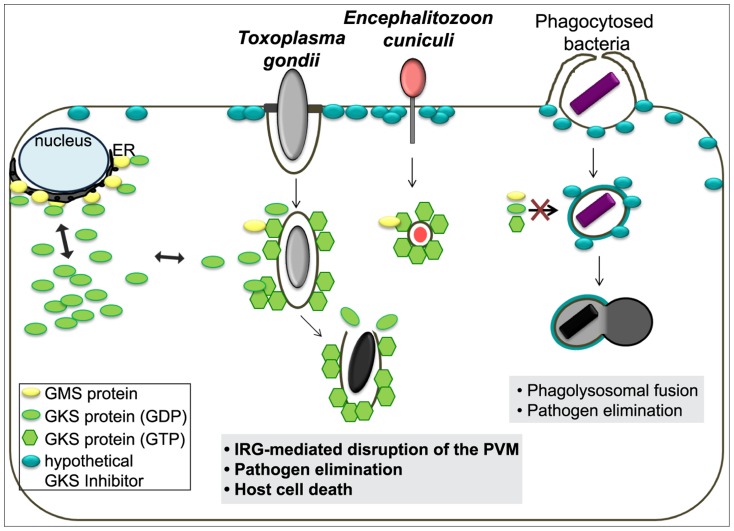
Scheme of the IRG resistance system and its target organisms. In IFNγ-stimulated mouse cells, GMS proteins localise mainly to endomembranes such as the ER and keep membrane-bound or cytosolic GKS proteins in a GDP-bound inactive state. Our current view is that the plasma membrane is protected by a hypothetical unknown factor that inhibits GKS protein-mediated damage. During host cell infection by *T. gondii* or *E. cuniculi*, invagination of the plasma membrane creates a parasitophorous vacuole that excludes the hypothetical factor and also does not carry GMS proteins. This “missing-self” allows GKS proteins to activate and accumulate on the PVM leading to the PV disruption, pathogen elimination and ultimately host cell death. However, bacteria entering via phagocytic mechanisms do not actively exclude the hypothetical factor and are therefore targeted for endolysosomal degradation.

In summary, every property of the IRG-dependent resistance mechanism that has been analysed for *T. gondii* is probably also valid against *E. cuniculi*, and, to the extent that it is known, also against *Chlamydia*. Since effective resistance dependent on IRG proteins seems to be perfectly correlated with the accumulation of IRG proteins on the parasitophorous vacuole, the challenge is to determine the common factor that enables IRG proteins to accumulate on the vacuoles of these three organisms but not on the vacuoles of other organisms. These three organisms cover three kingdoms of life: protozoa, bacteria, and fungi. The broad phylogenetic distribution makes it unlikely *a priori* that the IRG proteins target a common ligand expressed by all restricted pathogens on their vacuolar membranes. Our preferred view builds on a hypothesis first formulated by Martens [53] to account for the targeting of IRG proteins to the *T. gondii* vacuole rather than to other cellular organelles. Martens proposed the existence of a self-derived factor X expressed on the membranes of cellular organelles that inhibits the accumulation and activation of IRG proteins on these sites, thereby protecting these organelles from IRG protein mediated damage. Parasitophorous vacuoles, lacking factor X, would be exposed to IRG accumulation and activation. This elegant “missing self” model was confirmed some time later and “factor X” was revealed to be the three GMS proteins, Irgm1, Irgm2 and Irgm3, which are bound to distinct subsets of organellar membranes and act as guanine nucleotide dissociation inhibitors of the effector GKS proteins at these sites [16]. In the absence of one or more GMS proteins, GKS proteins form activated, GTP-bound assemblies in the cytoplasm, probably associated with “unprotected” organellar membranes [23], [41]. The GMS IRG proteins seem to fulfil exactly the role of Martens' Factor X for the distinction between intracellular organelles and a parasitophorous vacuole. However, GKS effector IRG proteins do not accumulate or activate on the plasma membrane, which to the best of our knowledge is not protected by any GMS protein. We are therefore forced to introduce a new hypothetical inhibitor associated with the plasma membrane that inhibits GKS activation at that location (Figure 8). Parasitophorous vacuoles are formed by invagination of the plasma membrane, and as we know with some precision from experiments with *T. gondii*, the vacuoles are receptive to IRG loading and activation immediately after parasite entry [18]. Thus entry of the parasite and formation of the parasitophorous vacuole must entail loss of the hypothetical plasma membrane-bound inhibitor. We propose that this is the essential distinction between those organisms that do, and those that do not, engage the IRG system, and that loss of the plasma membrane inhibitor is due to the unusual, non-phagocytic entry mechanisms of all three parasites.

Recently, the Atg5-dependent module of the ubiquitin-like conjugation system of autophagy has been proposed to mediate IRG targeting to the vacuole of *T. gondii* [19], [54], [55]. But since IRG and GBP proteins in Atg5^−/−^ cells form cytosolic GTP-bound aggregates [18], [19], [55] which are unable to target pathogens, loss of resistance appears rather to be caused by deregulated effector protein homeostasis. It has also been difficult to localise any autophagic component to *T. gondii* parasitophorous vacuoles [4]. Recent data from Choi et al. (2014) suggest occasional localisation of native LC3_II_ at the PVM, but on only a small minority of vacuoles, uncorrelated with the presence of IRG proteins [54].

The entry of *T. gondii* into cells has been studied in considerable detail and probably provides the best system for establishing the identity of the plasma membrane inhibitor. Several categories of protein are depleted from the developing vacuolar membrane, presumably as a result of the sieving action of the parasite-derived RON protein complex formed at the moving junction through which the parasite enters the cell [56], [57], [58], [59]. Apart from transient activation of host actin at the moving junction [60], [61], there is also no evidence that components of the cortical cytoskeleton remain associated with the nascent vacuole.

In the case of *Encephalitozoon cuniculi*, electron microscopic studies suggested that the PV membrane is derived from the host cell [29]. Subsequent studies from Bohne and colleagues established that the early PVM is non-fusogenic and devoid of any endolysosomal markers immediately after invasion [39], and moreover that the lipids of the PVM are indeed host cell-derived and that the PVM also forms simultaneously with the extrusion of the sporoplasm. This is all consistent with the suggestion that the early PVM is an invagination of the host cell plasma membrane [28], [62]. Due to the high speed of host cell entry, we suggest that physical forces may determine the presence and composition of host cell surface proteins on the newly formed PVM, which would be a prerequisite of IRG protein recognition. Although *E. cuniculi* spores are actively phagocytosed by macrophages, this is unlikely to be a biologically significant entry route, because it does not contribute to the intracellular meront population in mouse macrophages [63] or mouse embryonic fibroblasts (unpublished results Springer-Frauenhoff).

This study was designed based on knowledge of the interaction of the mouse IRG protein system and *T. gondii*. We found four major similarities in the action of the IRG system in defense against *E. cuniculi*: (1) the relocalisation of multiple IRG proteins to the cytosolic face of the PVM; (2) cooperativity by double-loading; (3) IFNγ- and infection- dependent host cell death and (4) IDO-independent IFNγ-mediated restriction in mouse cells.

While the IRG system clearly plays an essential role in defending mice and probably other small rodents against certain infections, there is every reason to believe that the system is effectively completely absent from humans and higher primates, birds, cats and doubtless many other mammalian species too [21]. The sporadic occurrence of a developed IRG system among vertebrate groups suggests that its possession is costly and only justified when certain classes of parasite exert intense selection pressures [14].

## Materials and Methods

### Ethics statement

All animal experiments were conducted under the regulations and protocols for animal experimentation according to the German “Tierschutzgesetz” (Animal Experimentation Law). The local government authorities, Landesamt für Natur- und Umweltschutz Nordrhein-Westfalen, and its ethics committee approved the work (LANUV Permit No. 84-02.05.40.14.004).

### Cell culture

Primary C57BL/6 mouse embryonic fibroblasts (MEFs) were prepared from mice at day 14 *post coitum*. *Irgm1^−/−^*, *Irgm3^−/−^*, *Irgm1/Irgm3^−/−^*, *Irgd* ^−/−^ MEFs (kindly provided by Greg Taylor) or *Irga6^−/−^*MEFs [42] were immortalized by transfection of pSV3-neo plasmid [64] and pPur (Clontech, Saint-Germain-en-Laye, France) in a ratio 9∶1 using FuGENE HD (Roche, Mannheim, Germany) according to the manufacturer's instructions. After 24 h, cells were put under selection with 3 µg/ml puromycin (Clontech). Primary and transformed MEFs as well as mouse rectal carcinoma CMT-93 cells (ATCC CCL-223) were cultured in DMEM, high glucose (Invitrogen Life Technologies, Darmstadt, Germany) supplemented with 10% fetal calf serum (FCS, Biochrom AG, Berlin, Germany), 2 mM L-glutamine, 1 mM sodium pyruvate, 1× MEM non-essential amino acids, 100 U/ml penicillin and 100 mg/ml streptomycin (all PAA, Pasching, Austria). Human foreskin fibroblasts (Hs27; ATCC CRL-1634) were cultured in IMDM, high glucose (Invitrogen Life Technologies) supplemented with 5% FCS, 100 U/ml penicillin and 100 mg/ml streptomycin (PAA). Cells were stimulated with 200 U/ml of mouse IFNγ (PeproTech, Rocky Hill, NJ, USA) for 24 h. For IDO-inhibition, L-tryptophan (W) (Sigma-Aldrich Co., Saint Louis, MO, USA) was added 15 min prior to infection.

### *In vitro* passage of *E. cuniculi* and infection of mouse fibroblasts

*E. cuniculi* spores were a generous gift from Prof. Peter Deplazes (University of Zürich, Switzerland). Spores were routinely propagated in Hs27 cells as described in [62]. Briefly, infected monolayers were scraped 7–12 days post infection and the suspension was passed through a 26G needle. The first centrifugation (10 min at 500 rpm) removed the host cell debris, whereas the second centrifugation (20 min at 2500 rpm) sedimented the spores. A stock solution with 4×10^7^ spores/ml PBS was stored at 4°C for max. 3 month. For infection assays, 8–12×10^4^ host cells were seeded in 6-well plates 48 h prior infection, optionally stimulated, and infected with a multiplicity of infection (MOI) of 10 parasites per host cell for microscopic assays and MOI 5 for Western Blot analysis. In order to obtain synchronous infection, spores were allowed to infect the cells for 2–4 h followed by one careful washing step with PBS and addition of fresh medium. Cells were fixed or harvested at the indicated time points post infection. For *E. cuniculi* genotyping, 60×10^7^ *E. cuniculi* spores were centrifuged for 20 min at 2500 rpm, resuspended in 200 µl PBS and 20 µl of Proteinase K, DNA was isolated with the DNeasy Blood & Tissue DNA purification kit (Qiagen, Hilden, Germany) according to manufactures instructions. The rRNA gene region of large (LSU rRNA) and small ribosomal subunit (SSU rRNA) and ITS region were amplified as described in [65]. The *E. cuniculi* stain used in this study had the genotype I.

### Immunological reagents

The following immunoreagents were used: rabbit anti-Irgm1 polyclonal antiserum (pAS) rbMAE15 [66], rabbit anti-Irgm2 pAS H53/3 [4], [18], rabbit anti-Irga6 pAS 165/3 [67], anti-Irga6 mouse monoclonal antibody (mAB) 10D7/10E7 [17], anti-Irgb6 mouse mAB B34 [68], anti-Irgb6 goat polyclonal antibody (pAB) A20 (sc-11079, Santa Cruz Biotechnology, Inc., Santa Cruz, CA, USA), anti-meront mouse mAB 6G2 [39], anti-SWP1 [69], anti-Calnexin rabbit (pAB) (Calbiochem Merck KGaA, Darmstadt, Germany). Secondary antibodies were Alexa Fluor 488/555/647-labeled donkey anti-mouse, -rabbit, and -goat antisera (all Molecular Probes, Invitrogen Life Technology), donkey anti-rabbit- (GE Healthcare, Freiburg, Germany), and goat anti-mouse-HRP (horseradish peroxidase) (Pierce, Thermo Fisher Scientific, Bonn, Germany) antisera. 4′, 6-Diamidino-2-phenylindole (DAPI, Roche, Mannheim, Germany) was used for nuclear staining at a final concentration of 0.5 mg/ml.

### Immunocytochemistry

Immunocytochemistry was carried out on paraformaldehyde-fixed cells grown on glass cover slips as described earlier [4]. In brief, cells were permeabilized and blocked with 3% BSA and 0.1% saponin (both Roth, Karlsruhe, Germany) in PBS, stained with the primary antibodies diluted in blocking buffer for 1 h at room temperature or overnight at 4°C following incubation with the secondary antibody diluted in blocking buffer for 30 min at room temperature. Between all steps cells were triple washed with 0.1% saponin in PBS and then mounted on glass microscopic slides in ProLong Gold anti-fade reagent (Invitrogen Life Technology). The images were taken with an Axioplan II fluorescence microscope and AxioCam MRm camera and processed by Axiovision 4.7 (all Zeiss, Oberkochen, Germany). All samples were counted blind.

### Live cell imaging

Live cell imaging was performed in μ-slide I chambers (Ibidi, Munich, Germany) as described earlier [20]. For live cell experiments, *wt* MEF cells were transiently transfected with pEGFP-N3-Irga6-ctag1 [20] using FuGENE HD (Roche) according to the manufacturer's instructions and induced with 200 U/ml IFNγ. After 24 hours cells were infected with *E. cuniculi* spores at a MOI 50 in phenol red-free RPMI 1640 (PAA). After infection with *E. cuniculi*, the cells were observed with a Zeiss Axiovert 200 M motorized microscope fitted with a wrap-around temperature-controlled chamber (Zeiss). The time-lapse images were obtained and processed by Axiovision 4.6 software (Zeiss).

### SDS-PAGE and western blot

At 2 or 5 days post infection, MEF or CMT-93 cells were washed with PBS once and directly lysed in 200 µl 2× SDS sample buffer (2% SDS, 100 mM Tris/HCl (pH 6.8), 10% Glycerol, 0.005% bromophenol blue, 1.4% β-mercaptoethanol). The lysates were transferred into Eppendorf tubes and boiled 5–10 min at 95°C. 15–20 µl were subjected to 10% SDS-PAGE and Western blot. Protein transfer was confirmed by staining the nitrocellulose membranes with Ponceau S solution [0.2% Ponceau S (Roth) and 3% acetic acid in dH_2_O]. Membranes were blocked in 5% non-fat dry milk in PBS and probed for the proteins of interest with the indicated primary and HRP-coupled secondary antibodies.

### Cell viability assay

Primary MEF cells (5000 cells/96-well) were seeded and induced with IFNγ for 24 h or left untreated. The cells were then infected with *E. cuniculi* spores at the indicated MOI for 24 h or 48 h. Thereafter, viable cells were quantified by the CellTiter 96 AQueous non-radioactive cell proliferation assay (Promega, Mannheim, Germany) according to the manufacturer's instructions. Infection with avirulent *T. gondii* Me49 served as positive control [20].

### Cell necrosis assay

MEF cells grown in 6 cm-dishes were induced with IFNγ for 24 h and infected with *E. cuniculi* spores at a MOI 10. At 24 h post infection, Bisbenzimide Hoechst 33342 and Propidium Iodide (both Sigma-Aldrich) were added to the medium (1 µg/ml final concentration for both) and incubated at 37°C for 15 min. 10 fluorescent pictures per sample were photographed with the Zeiss Axiovert 200 M microscope with a 10 fold magnification. Total cell number (Hoechst-positive nuclei) and dead cells (PI-positive nuclei) were automatically enumerated using the Volocity software (PerkinElmer, Santa Clara, CA, USA). At least 500 cells were counted per sample and percentage of dead cells per total cell number was calculated. In five independent experiments, a total of 10.000 cells or more was counted per sample.

## Supporting Information

Figure S1 **IRG proteins load onto the** ***E. cuniculi*** **PVM in a time-dependent manner.** MEFs were transiently transfected with Irga6-ctag1-EGFP and induced with IFNγ for 24 hours. Cells were infected with *E. cuniculi* spores at a MOI 50 and analyzed with time lapse video microscopy starting 5 h post infection (A) or 20 post infection (B). (**A**) The top panel shows the green fluorescent channel only, the arrow points at the Irga6-EGFP ring which seems to break up within the next 37 minutes. The white box shown as inset is the magnified area of interest. The panel below shows the corresponding phase contrast images. Transects were drawn through the meront (dashed white line) and the profiles below show the pixel intensity of IRG staining within this transect. Scale bar: 10 µm. (**B**) On the top, merged images of green fluorescence and phase contrast are shown for the start and end point of the time laps series. The magnified area within the white box of the green fluorescent channel only is shown below in the zoom in pictures as time series. Irga6 protein seems to accumulate as a ring-like structure within 20 minutes and then the level decreases again. Transects were drawn through the meront (white line marked by the arrow head) and the profiles below show the pixel intensity of IRG staining within this transect.
(TIF)
