# Correction: Identification of the Microsporidian *Encephalitozoon cuniculi* as a New Target of the IFNγ-Inducible IRG Resistance System

**DOI:** 10.1371/journal.ppat.1004590

**Published:** 2014-12-08

**Authors:** The PLOS Pathogens Staff

There are errors in the Authorship List, Citation, and Author Contributions sections.

The first listed author's name is spelled correctly, but the distinction between surname and given name is incorrect, which has resulted in an error in the citation. The author's surname is Ferreira-da-Silva and the author's given name is Marialice da Fonseca.

The correct citation is: Ferreira-da-Silva Mda F, Springer-Frauenhoff HM, Bohne W, Howard JC (2014) Identification of the Microsporidian Encephalitozoon cuniculi as a New Target of the IFNγ-Inducible IRG Resistance System. PLoS Pathog 10(10): e1004449. doi:10.1371/journal.ppat.1004449

In the Author Contributions section, Marialice da Fonseca Ferreira-da-Silva (MdFFdS) should be listed as one of the persons who conceived and designed the experiments.
