# Supplementary figures and images for: Identification of the Microsporidian Encephalitozoon cuniculi as a New Target of the IFNγ-Inducible IRG Resistance System
Source: PLoS Pathog. 2014 Oct 30;10(10):e1004449. doi: 10.1371/journal.ppat.1004449 (PMC4214799; doi:10.1371/journal.ppat.1004449)

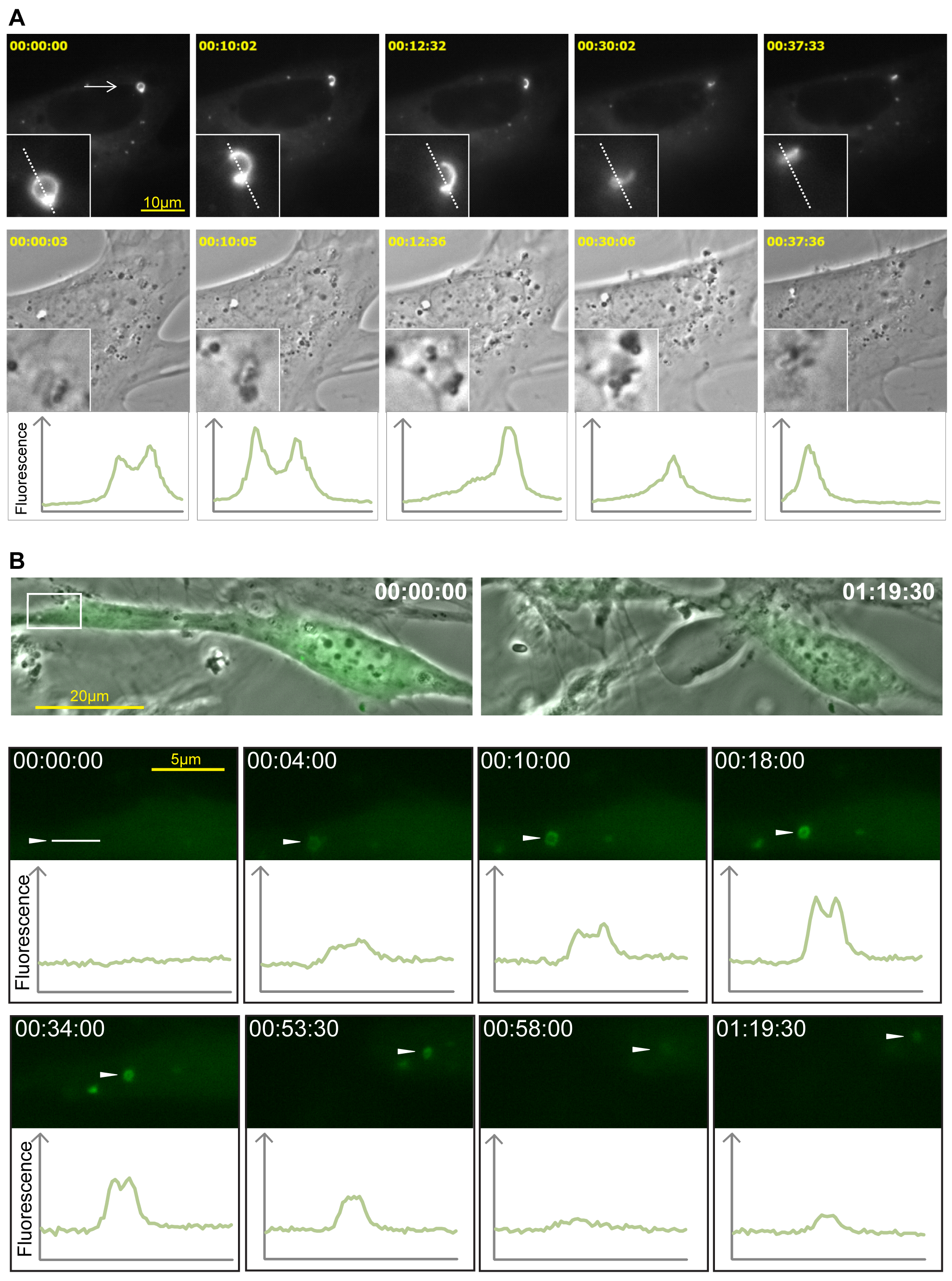

Supplement: Figure S1 — IRG proteins load onto the E. cuniculi PVM in a time-dependent manner. MEFs were transiently transfected with Irga6-ctag1-EGFP and induced with IFNγ for 24 hours. Cells were infected with E. cuniculi spores at a MOI 50 and analyzed with time lapse video microscopy starting 5 h post infection (A) or 20 post infection (B). (A) The top panel shows the green fluorescent channel only, the arrow points at the Irga6-EGFP ring which seems to break up within the next 37 minutes. The white box shown as inset is the magnified area of interest. The panel below shows the corresponding phase contrast images. Transects were drawn through the meront (dashed white line) and the profiles below show the pixel intensity of IRG staining within this transect. Scale bar: 10 µm. (B) On the top, merged images of green fluorescence and phase contrast are shown for the start and end point of the time laps series. The magnified area within the white box of the green fluorescent channel only is shown below in the zoom in pictures as time series. Irga6 protein seems to accumulate as a ring-like structure within 20 minutes and then the level decreases again. Transects were drawn through the meront (white line marked by the arrow head) and the profiles below show the pixel intensity of IRG staining within this transect. (TIF) [file ppat.1004449.s001.tif]
